# Anxiety about COVID-19 Infection, and Its Relation to Smartphone Addiction and Demographic Variables in Middle Eastern Countries

**DOI:** 10.3390/ijerph182111016

**Published:** 2021-10-20

**Authors:** Mohammad Farhan Al. Qudah, Ismael Salamah Albursan, Heba Ibraheem Hammad, Ahmad Mohammad Alzoubi, Salaheldin Farah Bakhiet, Abdullah M. Almanie, Soltan S. Alenizi, Suliman S. Aljomaa, Mohammed M. Al-Khadher

**Affiliations:** 1 Department of Psychology, College of Education, King Saud University, Riyadh 11451, Saudi Arabia; malqudah@ksu.edu.sa (M.F.A.Q.); ibursan@ksu.edu.sa (I.S.A.); Soltan1427h@hotmail.com (S.S.A.); jomaa@ksu.edu.sa (S.S.A.); Alkhader2011@gmail.com (M.M.A.-K.); 2 Department of Psychology, AL Balqa Applied University Princess Alia College, Amman 11821, Jordan; Hammad.he@Yahoo.com; 3 Department of Psychology, College of Humanities and Sciences, Ajman University, Ajman 364, United Arab Emirates; A1970ahmad@hotmail.com; 4 Department of Special Education, College of Education, King Saud University, Riyadh 11567, Saudi Arabia; 5 Department of Educational Administration, College of Education, King Saud University, Riyadh 11451, Saudi Arabia; aalmana@ksu.edu.sa

**Keywords:** anxiety about COVID-19 infection, COVID-19, frequency, age, smartphone addiction, Middle Eastern countries

## Abstract

This study explores the level and frequency of anxiety about COVID-19 infection in some Middle Eastern countries, and differences in this anxiety by country, gender, workplace, and social status. Another aim was to identify the predictive power of anxiety about COVID-19 infection, daily smartphone use hours, and age in smartphone addiction. The participants were 651 males and females from Jordan, Saudi Arabia, the United Arab Emirates, and Egypt. The participants’ ages ranged between 18 and 73 years (M 33.36, SD = 10.69). A questionnaire developed by the authors was used to examine anxiety about COVID-19 infection. Furthermore, the Italian Smartphone Addiction Inventory was used after being translated, adapted, and validated for the purposes of the present study. The results revealed that the percentages of participants with high, average, and low anxiety about COVID-19 infection were 10.3%, 37.3%, and 52.4%, respectively. The mean scores of anxiety about COVID-19 infection in the four countries were average: Egypt (M = 2.655), Saudi Arabia (M = 2.458), the United Arab Emirates (M = 2.413), and Jordan (M = 2.336). Significant differences in anxiety about COVID-19 infection were found between Egypt and Jordan, in favor of Egypt. Significant gender differences were found in favor of females in the Jordanian and Egyptian samples, and in favor of males in the Emirati sample. No significant differences were found regarding workplace and social status. The results also revealed a significant positive relationship between anxiety about COVID-19 infection, daily smartphone use hours, and age on the one hand, and smartphone addiction on the other. The strongest predictor of smartphone addiction was anxiety about COVID-19 infection, followed by daily use hours. Age did not significantly contribute to the prediction of smartphone addiction. The study findings shed light on the psychological health and cognitive aspects of anxiety about COVID-19 infection and its relation to smartphone addiction.

## 1. Introduction

Epidemics are considered to be a prominent source of psychological and social disorders, e.g., fear, anxiety, and reluctance to communicate with others [1]. It is common during epidemics and pandemics for people to suffer from stress and anxiety, including the fear of infection and death, avoiding receiving medical treatment at health facilities, fearing the loss of relatives, and fearing isolation because of quarantine, which causes boredom, loneliness, and depression [2]. There is a psychoneurotic connection between acute inflammations of the respiratory system and psychological disorders, as occurred with SARS decades ago. People in quarantine suffer from boredom, anger, and loneliness. Symptoms such as cough and fever can increase anxiety, intrusive thoughts, and the fear of COVID-19 infection [3]. The world is currently experiencing the COVID-19 pandemic that spread to all countries in a short amount of time. The WHO declared the novel coronavirus outbreak a pandemic in March 2020, and predicted it to spread to all countries, urging countries to take the necessary steps to control it [4].

The number of people who have had a coronavirus infection is in the millions. It is, therefore, a threat that invokes anxiety, depression, and indignation in people. To protect themselves, people now adhere to social distancing so as to not catch the infection from close contact with others [5]. Being at home all the time can affect the mental health of both children and adults. Children and adolescents have therefore been advised to focus on home activities to forget about the negative effects of the coronavirus [6].

Middle Eastern countries have also been affected by the pandemic. By 7 July 2020, 214,000 confirmed coronavirus cases and 1968 deaths had occurred in Saudi Arabia. In the United Arab Emirates, 520,068 infected cases and 324 deaths were reported. In Egypt, 760,222 infected cases and 30,422 deaths were reported. A total of 1167 infected cases and 10 deaths were reported in Jordan [7]. Several studies have reported on the negative effects of epidemics and pandemics on the psychology of infected people and their caregivers [8,9]. Those studies reported a high level of psychological stress among people providing care to infected cases. In many studies, people with acute respiratory syndromes (Ebola, MERS, and SARS) were reported to have psychological disorders such as anxiety, depression, and other forms of mental illness [10,11,12]. In the Saudi context, in terms of the effect of MERS on psychological stress, female students had a higher level of psychological stress than that of male students [13]. In the Omani and Bahraini contexts, the coronavirus-induced anxiety among families was average, and no significant differences were found by country. However, there was a significant difference in favor of females, people aged over 40 years, people with lower educational levels, and unemployed people. Retired people were reported to experience the lowest level of anxiety [14].

In terms of the psychological impact, depression, anxiety, and stress at the beginning of the coronavirus pandemic in a sample of 1210 participants from 194 Chinese cities, 53.8% of the participants suffered an acute psychological impact because of the pandemic, whereas about 28.8% of the participants were found to suffer from average to acute anxiety [15]. In a study conducted in Italy, the percentage of people having high and severe coronavirus-related anxiety ranged between 2.89% and 7.43% [16]. The WHO also asserted that some populations, such as people working in health and security, had infection fears and suffered from stress due to dealing with infected people, work pressure, and changed sleep and eating routines. Ministers and leaders in authorities confronting the pandemic suffer from similar psychological effects [17]. Two surveys were also conducted by the British Academy of Medical Sciences via the Internet. The results of the first survey revealed that the majority of the sample suffered from problems with mental health. Participants reported fears about their health and access to support and services during the pandemic. The second survey reported anxiety among participants about social isolation and economic difficulties resulting from the pandemic. With expectations of the increased occurrence of anxiety and stress during the pandemic, researchers expect an increase in the number of depressed people and people who are prone to commit suicide. In 2003, during the SARS epidemic, the rate of suicide in people over 65 years witnessed a 30% increase. Researchers asserted that actions taken at that time to eliminate the spread of SARS had serious effects on people’s mental health, as unemployment rates and feelings of financial insecurity and poverty increased [18].

Research results concerning gender differences in epidemic-related anxiety are inconsistent, with some studies reporting higher levels of anxiety among females [15,16,19,20] and others reporting higher levels of anxiety among males [21]. Some studies have reported differences in anxiety about the future by gender in favor of females, and by social status in favor of the unmarried. No differences were found by profession [22,23,24]. Low-to-average anxiety levels were found between participants. The frequency of mild, average, and severe anxiety among participants was 7.7–78.8%, 5.6%, and 2.7–5.2%, respectively. The study did not find gender differences in depression and anxiety. On the other hand, it found differences in favor of the unmarried [25]. The authors in [15] found higher levels of anxiety among students than those among employeesworking personnel. The level of anxiety did not correlate with social status, the size of the family, or age. Similarly, social status, having no children, and workplace did not significantly contribute to anxiety or depression [16].

Smartphone use has been globally widespread during the coronavirus pandemic, which has induced feelings of isolation, social distancing, and a need for leisure, recreation, and shopping [26]. With this intensive use, smartphone addiction has become a universal concern [27]. It is a recent phenomenon in human behavior that can adversely affect the mental health and social functioning of people who overuse smartphones [28]. Smartphone addiction is the overuse or compulsive use of smartphones, resulting in negative consequences in social, behavioral, and emotional functioning [29]. It is a form of behavioral addiction that makes the individual unable to control the strong desire to use the smartphone and its applications, with the loss of productivity, the denial of negative effects, preoccupation, and feelings of annoyance and even panic when deprived of the smartphone [30]. Some studies have shown a connection between smartphone addiction and psychological adjustment problems, e.g., anxiety and depression. A Korean study found that smartphone addiction can be predicted by depression [31]. A similar finding was also reached in a Chinese study [32], where loneliness, which relates to depression, was found to be a strong predictor of smartphone addiction. In an American study, social interaction anxiety was found to predict smartphone addiction [33]. Another study found a positive correlation between anxiety and depression and smartphone overuse [34]. Smartphone addiction could be predicted by anxiety and depression. Anxiety as a major symptom of smartphone addiction emerges once the person is deprived of their smartphone [35,36]. This shows that the smartphone itself is a source of anxiety [37]. Smartphone overuse is a factor leading to mental health problems. They also found that gender was the strongest predictor of depression. Symptoms of anxiety were more frequent in younger people [38]. A positive correlation between smartphone addiction and psychological stress was also found. Research also revealed a weak relationship between age and hours of use on the one hand, and smartphone addiction on the other [39].

More than one study did not find a correlation between age and smartphone addiction [40,41]. Meanwhile, a positive correlation was found between daily use hours and the problematic use of smartphones [42]. However, differences in smartphone addiction in favor of individuals using a smartphone for more than four hours a day were found [43]. This same finding was reported by Haug, who reported a correlation between smartphone addiction and daily use hours [44]. Facebook addiction and state anxiety could be predicted by an increased use rate. The interaction of gender and trait anxiety predicted Facebook addiction [45].

As mentioned above, and the increase in cases of COVID-19 infection around the world in general and in Middle Eastern countries in particular can lead to increased levels of anxiety with negative behavioral effects, such as smartphone addiction. The present study aimed to identify the level and frequency of anxiety about COVID-19 infection in some Middle Eastern countries, and differences in this anxiety by country, gender, workplace, and social status. The study also aimed to identify the predictive power of anxiety about COVID-19 infection variables, daily smartphone use hours, and age in smartphone addiction.

## 2. Method

### 2.1. Participants

This study comprised a total of 651 participants (222 males and 429 females representing 34.1% and 65.9%, respectively) from four Middle Eastern countries: Jordan (*n* = 271, 41.6%), Saudi Arabia (*n* = 179, 27.5%), the United Arab Emirates (*n* = 108, 16.6%), and Egypt (*n* = 93, 14.3%). Their age ranged between 18 and 73 years (M = 33.35, SD = 10.69). Of the 651 participants, 246 (37.7%) were single, 378 (58.1%) were married, and 27 (4.22%) were divorced. The number of participants working for the government, the private sector, and students were 242 (37.2%), 243 (37.3%), and 166 (25.5%), respectively.

### 2.2. Instruments

#### 2.2.1. Anxiety about COVID-19 Infection Scale

The authors developed a scale to measure anxiety about COVID-19 infection. To develop the scale, the authors surveyed scales in the relevant literature, e.g., the State-Trait Anxiety Inventory [46] and scales of social anxiety and general anxiety [15,47,48,49,50,51]. The authors also used anxiety indicators, including the WHO’s reports about prevention and the health guidelines for dealing with the virus. The scale had 40 items with 5-point Likert scales ranging from 5—‘to a very high degree’ to 1—‘to a very low degree’. The preliminary version of the scale was face-validated by five professors who specialized in psychology, measurement, and evaluation. They were asked to judge if items represented the measured trait, and if the wording of items was sound and clear. This resulted in modifying some items, but no deletions were made.

Correlations among items and the total score were computed. These ranged from 0.628 to 0.842, all of which were high and statistically significant. The unilaterality of the scale was established by factor analysis. The results revealed that all items were significantly loaded on the first factor. The first eigenvalue was 22.025, and the second eigenvalue was 2.345. The explained variance of the first factor was 55.63%. This is consistent with Rechase’s [52] suggestion that the unilaterality condition is met if the first factor can explain at least 20% of total variance. The reliability of the scale was then checked by computing the alpha Cronbach coefficient of participant scores. The scale yielded an alpha coefficient of 0.978, which indicates that the scale was highly reliable.

Participant scores on the scale ranged between 40 and 200. Scores were categorized by range into high anxiety (146.8–200) with a weighed mean ranging from 3.67 to 5, average anxiety (93.4–146.7) with a weighed mean ranging from 2.34 to 3.66, and low anxiety (40–93.3) with a weighed mean ranging from 1 to 2.33.

#### 2.2.2. Smartphone Addiction Inventory

After surveying the literature on smartphone addiction and the instruments used in relevant studies, we used the Smartphone Addiction Inventory (SPAI) that was used in the studies of Pavia, Cavani, Blasi, and Giordano [53] and Lin et al. [54]. It is an inventory developed on the basis of the Chinese Internet Addiction inventory (CIAS) [55]. Items of this inventory assess several dimensions of smartphone addiction: compulsory use, withdrawal, tolerance, and problems in relationships with others, and time and health management. The reliability examination of the inventory was originally performed on a Chinese sample of 283 university students. Another examination of its psychometric characteristics and factor structure was performed in Italy [53]. The sample consisted of 485 male and female students whose ages ranged between 10 and 27 years. Exploratory and confirmatory factor analyses revealed that the items of the inventory were loaded on five factors: time spent, compulsivity, daily life interference, craving, and sleep interference. The alpha Cronbach reliability coefficient of the whole inventory was 0.94.

The English version of the inventory was translated into Arabic by two bilingual researchers. The accuracy of translation was verified by back translation, which was performed by a third researcher. The retranslated version was then compared with the original English version, and differences were very few. Very few adaptations were made to make the inventory suitable to the Arab environment. As a result, the version used in the study originally had 24 items, measuring 5 dimensions with a 4-point rating scale ranging from 4—‘strongly agree’ to 1—‘strongly disagree’. Thus, a respondent’s score on the inventory ranged from 24 to 96. The higher the score of a respondent was, the higher their level of smartphone addiction.

The inventory was validated by having it refereed by specialists and by establishing its construct validity. For construct validation, correlations among items and the total score were computed, and they ranged between 0.63 and 0.85, which were all statistically significant. The unilaterality of the inventory was established by exploratory factor analysis. The results revealed that all items significantly loaded on the first factor. Eigenvalues were 12.632 for the first factor and 1.460 for the second factor. The explained variance of the first factor was 52.635 (90% of the total variance before rotation) and 31.299 (53% of the total variance after rotation). This indicates that the inventory was unilateral. The reliability of the inventory was then checked by computing the alpha Cronbach coefficient of the participants’ scores. The inventory yielded an alpha coefficient of 0.982, which indicates that it was highly reliable.

### 2.3. Procedures

The authors developed an electronic questionnaire, including the scale on anxiety about COVID-19 infection, the smartphone addiction inventory, and demographic data. The link to the questionnaire was then sent to participants via WhatsApp (Facebook Inc, Menlo Park, CA, USA) and Twitter with the help of authors who live in the four countries included in the study. Completion of the questionnaire took three weeks (the last two weeks of May and the first week of June 2020). The application of the questionnaire coincided with the application of strict health procedures, imposing social distancing and quarantining. Movement between cities was also prohibited in the four countries. Other procedures included the prohibition of gatherings, distant learning, school closures, and restricted travel. The aims of the study and instructions for completing the questionnaire were provided with the electronic questionnaire. Participants were told that the completion of the questionnaire was voluntary, and that data collected from the completed questionnaires would only be used for research purposes. For this reason, they were not required to write their names or give any information about their identities. They were also told that the honest completion of the questionnaire would be the key for the successful completion of the study. Following this, the authors scored and codified the received completed questionnaires, and categorized the data according to the study variables.

### 2.4. Data Analysis

The obtained data were statistically analyzed using IBM SPSS Statistics-25 (IBM, Armonk, NY, USA).

To answer the research question about the frequency of anxiety about COVID-19 infection, descriptive measures (frequencies, percentages, means, and standard deviations) were used. The *t*-test for independent samples was used to identify gender differences in anxiety about COVID-19 infection, and the ANOVA test was used to identify differences in anxiety about COVID-19 infection by country, social status, and workplace. Pearson’s correlation was used to explore relationships among variables. Lastly, the multiple stepwise regression test was used to explore the predictive power of the anxiety about COVID-19 infection scale, daily smartphone use hours, and age in smartphone addiction.

## 3. Results

### 3.1. Frequency of Anxiety about COVID-19 Infection among Participants

Table 1 shows means, standard deviations, and percentages of anxiety about COVID-19 infection by country.

Table 1 shows that the country with the highest anxiety about COVID-19 infection was Egypt (M = 106.22), followed by Saudi Arabia (M = 98.31), the United Arab Emirates (M = 96.53), and Jordan (M = 93.45).

### 3.2. Differences among Countries in Anxiety about COVID-19 Infection

To identify differences among the four countries in anxiety about COVID-19 infection, the ANOVA test was performed. These results are listed in Table 2.

The data in Table 2 reveal that there were significant differences among countries in anxiety about COVID-19 infection (*p* = 0.033, a < 0.05). The effect size was partial eta squared = 0.13. The country variable explained 13% of variance in anxiety about COVID-19 infection. After performing post hoc analysis using the Scheffe test, differences were found to be significant only between Jordan and Egypt (*p* = 0.037, a < 0.05) in favor of Egypt, of which the mean was higher.

### 3.3. Gender Differences in Anxiety about COVID-19 Infection

The *t*-test was performed to explore gender differences in anxiety about COVID-19 infection in the four countries. Table 3 presents these results.

Table 3 shows that there were no statistically significant gender differences (a = 0.05) in anxiety about COVID-19 infection in Saudi Arabia. However, there were significant differences in Jordan (*p* = 0.007, a < 0.05) and Egypt (*p* = 0.018, a < 0.05) in favor of females, and in the United Arab Emirates (*p* = 0.013, a < 0.05) in favor of males. The effect size according to Cohen was small in the Jordan sample (0.363), and average in the Egyptian (0.550) and Emirati (0.626) samples. At the level of the whole sample, there were significant differences (*p* = 0.013, a < 0.05) in favor of females with a low effect size (0.206).

### 3.4. Differences in Anxiety about COVID-19 Infection by Social Status

Differences in anxiety about COVID-19 infection by social status were explored by performing the ANOVA test with three categories: single, married, and divorced. These results are presented in Table 4.

Table 4 shows that there were no significant differences (*p* = 0.364, a < 0.05) in anxiety about COVID-19 infection by social status.

### 3.5. Differences in Anxiety about COVID-19 Infection by Workplace

Differences in anxiety about COVID-19 infection by workplace were explored by performing the ANOVA test with three categories: governmental job, private-sector job, and student. These results are presented in Table 5.

Table 5 shows that there were no significant differences (*p* = 0.390, a < 0.05) in anxiety about COVID-19 infection by workplace.

### 3.6. Predicting Smartphone Addiction by Anxiety about COVID-19 Infection, Daily Smartphone Use Hours, and Age

Pearson correlations among study variables were computed. A statistically significant (a = 0.01) negative relationship (r = −0.122) was found between age and smartphone addiction. A statistically significant (a = 0.05) negative relationship (r = −0.071) was found between age and anxiety about COVID-19 infection. A statistically significant negative relationship (r = −0.242) was found between age and daily smartphone use hours. Lastly, a statistically significant (a = 0.01) positive relationship (r = 0.427) was found between smartphone addiction and anxiety about COVID-19 infection, and between smartphone addiction and daily smartphone use hours (r = 0.357).

To identify the predictive power of anxiety about COVID-19 infection, daily smartphone use hours and age in smartphone addiction, stepwise multiple regression was used. Table 6 shows these results. Collinearity was checked using the variance inflation factor (VIF), and the value was less than 10 (average VIF = 1), which indicated that the problem of multicollinearity was not present.

Table 6 reveals that smartphone addiction can be predicted by anxiety about COVID-19 infection and daily smartphone use hours but not by age (R^2^ = 0.183 for step 1, F (1.649) = 144.972, *p* < 0.001); for step 2, ΔR2 = 0.090, F (2.648) = 121.635, *p* < 0.01).

Anxiety about COVID-19 infection was the best predictor of smartphone addiction, as it could explain 0.181 of the variance in smartphone addiction. The interaction of anxiety about COVID-19 infection and daily smartphone use hours explained 0.273 of the variance in anxiety about COVID-19 infection. Thus, the daily use hours variable could predict an additional amount of smartphone addiction of 0.090, which was significant at the 0.01 level. The prediction equation can be stated as follows: smartphone addiction = 31.160 + 0.168 × anxiety about COVID-19 infection + 1.127 × daily use hours.

## 4. Discussion

The results of the study revealed that the percentages of participants who had high, average, and low anxiety about COVID-19 infection were 10.3%, 37.3%, and 52.4%, respectively. This refers to an average level of anxiety at the level of the whole sample. Regarding the frequency of anxiety in the four target countries, all frequencies were at the average level, with Egypt being in first place with a mean of 2.655, followed by Saudi Arabia (M = 2.458), the United Arab Emirates (M = 2.4130), and Jordan (M = 2.336). This finding largely concurs with the findings of previous studies conducted in some Gulf states during the outbreak of the pandemic. Those studies reported average anxiety and stress resulting from the pandemic [13,14]. The percentages here were also close to their counterparts in the Chinese study [15], and slightly higher in high anxiety than the percentages in the Italian study [16]. The current percentages, showing average and high anxiety, exceeded their counterparts in the Australian study [25]. Data collection in the present study coincided with the application of strict health procedures in all countries globally, e.g., the prohibition of gatherings and curfews.

Regarding differences in the frequency of anxiety about COVID-19 infection in the four Arab countries, the results revealed significant differences between Egypt and Jordan in favor of Egypt. This finding seems logical given that Egypt, in comparison with Jordan, was late in imposing health restrictions and giving the real numbers of infected cases. Unlike Egypt, Jordan took actions with the appearance of the first infected case. Jordan imposed a curfew and closed schools, governmental institutions, mosques, and airports. Such procedures largely reduced the number of infected cases. Accordingly, the number of infected cases in Jordan up to 7 July was 1167, with a recovery rate of 82% and a death rate of 0.08%. On the other hand, the number of infected cases in Egypt up to 7 July was 76,222, with a recovery rate of 28% and death rate of 4.5%. This may refer to a deficiency in health procedures and the provision of health support to critical cases that required special and costly treatment protocols. Egypt’s population is also more than 100 million. The frequency of high anxiety (15.1%) in Egypt exceeded its counterparts in a number of Arab and Asian countries that were covered in previous studies [13,14,15,16,25].

The finding of insignificant differences in the level of anxiety about COVID-19 infection between Saudi Arabia and the United Arab Emirates is in line with the study conducted on Omani and Bahraini samples [14], in which no significant differences were found between the two countries in anxiety about COVID-19 infection. The Saudi environment is largely similar to the Omani and Bahraini environments.

Analysis of the data collected from the whole sample revealed gender differences in anxiety about COVID-19 infection in favor of females. Gender differences were also found in three of the four countries. The differences were in favor of females in the Egyptian and Jordanian samples, and in favor of males in the Emirati samples. No gender differences were found in the Saudi sample. This general finding about females having higher anxiety about COVID-19 infection than males concurs with several previous studies [13,15,16,56]. This finding is also consistent with previous studies exploring gender differences in general psychological anxiety [19,20,22,23,24,57].

The finding about gender differences in COVID-19 infection anxiety in favor of males in the Emirati sample can be explained by the fact that most respondents in the sample were non-Emirati, who represent about 89% of the total population in the United Arab Emirates. Those respondents live with their families and work in various sectors in the country. Male residents in the United Arab Emirates may have higher levels of anxiety than females do because of fears about their jobs with the economic damages resulting from the pandemic. Some sectors there made some employees redundant or reduced their salaries. For this reason, non-Emirati male employees may fear the loss of their jobs and becoming unable to sustain their families. Women, on the other hand, do not have these fears because women in Eastern societies are not required to work and sustain their families. Furthermore, women stay at home most of the time, which makes them less anxious about catching the infection. This finding is consistent with [14], in which residents had higher levels of anxiety because of the lack of occupational security and being in countries other than their own. This finding is also consistent with studies investigating general anxiety and anxiety about the future, in which males were reported to have higher levels of anxiety than females [21,58].

Males and females in the Saudi sample had comparable levels of anxiety about infection. A possible explanation for this finding is that they live in the same environment and face the same threats. This finding is in line with the Australian study, in which no significant difference in infection anxiety was found [25].

Regarding the effect of social status on anxiety about infection, no significant differences were found among single, married, and divorced participants. This means that anxiety about infection is not affected by one’s social status, or being single, married, or divorced. This same finding was reached in [15,16]. It is, however, inconsistent with [25], in which the unmarried had a more significant level of anxiety. This finding also concurs with studies conducted before the coronavirus pandemic, in which the unmarried had higher levels of general anxiety [22,23,24].

As with social status, no gender differences were found in infection anxiety by workplace. Participants working for the government and the private sector and students had comparable levels of anxiety about infection. This finding is consistent with [16], and with studies that did not find differences in general anxiety by workplace [22,23,24]. However, it is inconsistent with [15], in which students outnumbered employees in terms of anxiety, and with [14], in which unemployed respondents outnumbered employees in terms of infection anxiety. Overall, social status and workplace need to be further studied with other variables such as educational level, income, and age because of the inconsistent results about the latter two variables in the few studies conducted so far.

Regarding the predictive power of infection anxiety, daily smartphone use hours, and age in smartphone addiction, stepwise multiple regression analysis revealed that smartphone addiction can be predicted by infection anxiety and daily smartphone use hours. Age, on the other hand, did not contribute to the prediction of smartphone addiction. Infection anxiety was the strongest predictor of smartphone addiction, followed by daily use hours. This means that people who are more anxious about infection tend to excessively use their smartphone. The authors did not find studies exploring the relationship between infection anxiety and smartphone addiction. However, the current study’s findings are in line with previous studies that reported a positive correlation between general anxiety, depression, stress, and loneliness on the one hand, and smartphone addiction on the other [9,27,28,29,30,31,32,33,34,37,39].

This finding seems logical and concurs with the mainstream views from previous research. With the outbreak of the coronavirus, and restrictions such as social distancing and staying at home most of the time, the smartphone can be the only resort for people in order to vent, pass time, and search for information about the virus. The smartphone is also used for distant learning due to the closure of schools. People may therefore excessively use the smartphone to the degree that they cannot control the time spent in its use. This, in turn, can lead to compulsivity, sleep interference, and excessive attachment to the smartphone, which are all symptoms of addiction. This concurs with [35,36], reporting anxiety as a major symptom of smartphone addiction. It also concurs with the assertion of [59] that overdependence on smartphones and the use of social media to know about current events can result in the fear of missing events, known as “fear of missing out”. The finding that daily use hours contribute to smartphone addiction is consistent with some studies [42,43,44,45], and is inconsistent with [39], in which a weak relationship was found between smartphone use hours and addiction.

The finding that age did not contribute to the prediction of smartphone addiction despite the presence of a significant negative relationship between them indicates that age is not a factor contributing to smartphone addiction during the coronavirus pandemic. This is in line with most studies that have examined the relationship between age and smartphone addiction [39,40,41].

Lastly, Pearson’s correlation revealed a significant, weak relationship between age and infection anxiety. This finding is partly in line with studies in which anxiety was found to be more frequent among young people [16,25,56]. It also concurs with the contention that young people are more prone to anxiety because of their quick access to information via social media [60]. This finding is inconsistent with [14], in which people aged over 40 years were found to be more anxious about infection, and with [15], in which age did not correlate with anxiety.

## 5. Conclusions

This study explored anxiety about COVID-19 infection and its relationship with some psychological and demographic variables. It revealed that this anxiety exists in some Middle East countries. Regardless of their social status, workplace, and age, participants in the study suffered average-to-high infection anxiety. Some sort of intervention is therefore required so this anxiety does not become morbid. The study also revealed that infection anxiety can lead to smartphone addiction, with all its negative psychological and physical effects, as well as the disorder known as nomophobia. Women were found to be more anxious about infection. Anxiety about infection and daily use hours were found to significantly contribute to smartphone addiction. It is therefore necessary to develop preventive programs to eliminate this phenomenon. Awareness must also be raised about the judicious use of smartphones. People should be advised on how to find alternative ways to fruitfully spend time, control their desire to use smartphones, and sterilize their phones, which can be a source of infection. It is also recommended that social media should be used to support people during the pandemic, and instruct them on how to keep safe and manage their anxiety. They should be told not to show fear and anxiety in front of their children, as this can leave negative effects on their development. People, especially mothers, should be advised not to spend too much time on social media, as this can make them anxious. This can adversely affect childcare, which might cause insecure attachment. During quarantine, people should telecommunicate with relatives to alleviate the impact of social isolation on children and adolescents. People can make an opportunity out of the crisis to practice activities and hobbies for which they previously had no time. Adults should distract children and adolescents from bad news by providing them with daily home activities and events. The use of smartphones and electronic games by children should be monitored so that they do not become addicted to them.

It is a good idea to develop electronic programs to enhance people’s psychological hardiness and to teach them how to face crises. There should also be programs of interest for old people to help them safely pass the time. It is also necessary to use social media to spread awareness about the virus and preventive healthcare. In this respect, medical sites do not make good use of social media.

Study results show that further research is required to explore anxiety about infection on larger samples and different populations. Future research is expected to focus on the negative effects of infection anxiety. Research endeavors are also required to develop and test the effectiveness of counseling and preventive programs in eliminating pandemic-related anxiety. Researchers can also examine the relationship between anxiety about infection and other variables—such as depression, burnout, anxiety about the future and death, psychological security, hardiness, optimism and pessimism, healthy behavior, self-efficacy, and achievement—and attitudes to the vaccination process.

Even though the results of the study documented the relationship between smartphone addiction and gender, the application of the instruments to a limited sample from four Middle Eastern countries ,whose ages ranged between 18 and 73, limits the generalizability of the results to age groups and populations in different contexts. Furthermore, the study was limited to an electronic questionnaire distributed via social media (WhatsApp and Twitter) during the period of restrictions on movement and social distancing. The authors used the available data in the four countries about the numbers of infected cases and deaths up to 7 July 2020. Cautious interpretation of results is important due to the use of a self-reported questionnaire. The results of self-reported questionnaires are prone to be affected by social desirability. Lastly, in this study we used the descriptive–comparative method. Further experimental and longitudinal studies using quantitative and qualitative data collection tools are required.

## Figures and Tables

**Table 1:** Means, standard deviations, and percentages of anxiety about COVID-19 infection by country.

High ACI ** %	Medium ACI ** %	Low ACI ** %	SD	Mean *	*n*	Country
22 −8.20%	92 −33.90%	157 −57.90%	34.8	93.45	271	Jordan
21 −11.70%	69 −38.50%	89 −49.70%	37.8	98.31	179	Saudi Arabia
10 −9.20%	42 −38.90%	564 −51.9	37.3	96.53	108	United Arab Emirates
14 −15.10%	40 −43.00%	39 −41.90%	36.6	106.22	93	Egypt
67 −10.30%	243 −37.30%	341 −52.40%	36.48	97.12	651	Total

* total score = 1. ** Anxiety about COVID-19 infection.

**Table 2:** Differences among countries in anxiety about COVID-19 infection.

Effect Size	sig	*f*-Value	Mean Squares	df	Sum of Squares	Source of Variance	Variable
0.13	0.033	2.938	3875.175	3	11,625.52	Between groups	Country
			1319.128	647	853,475.9	Within groups	
				650	865,101.4	Total	

**Table 3:** *t*-test for gender differences in anxiety about COVID-19 infection.

Effect Size	Sig	*t*-Value	SD	Mean	*n*	Gender	Country
0.363	0.007	2.737	31.624	84.69	81	Male	Jordan
			35.527	97.19	190	Female	
-	0.088	1.713	37.828	93.73	94	Male	Saudi Arabia
			37.263	103.36	85	Female	
0.626	0.013	2.528	34.366	115.1	20	Male	U.A.E
			36.825	92.31	88	Female	
0.55	0.018	2.405	31.043	92.3	27	Male	Egypt
			37.39	111.91	66	Female	
0.206	0.013	2.494	35.33	92.18	222	Male	Total
			36.845	99.68	429	Female	

**Table 4:** ANOVA for differences in anxiety about COVID-19 infection by social status.

sig	*f*-Value	Mean Squares	df	Sum of Squares	Source of Variance	Variable
0.364	1.011	1345.536	2	2691.071	Between groups	Marital status
		1330.88	648	862,410.3	Within groups	
			650	865,101.4	Total	

**Table 5:** ANOVA for differences in anxiety about COVID-19 infection by workplace.

sig	*f*-Value	Mean Squares	df	Sum of Squares	Source of Variance	Variable
0.39	0.942	1253.91	2	2507.821	Between groups	Working position
		1331.163	648	862,593.6	Within groups	
			650	865,101.4	Total	

**Table 6:** Stepwise multiple regression for the predictive power of anxiety about COVID-19 infection, daily smartphone use hours, and age in smartphone addiction.

sig	T	β	Std. Error	B	Variable
Step1					
<0.001	23.053		1.607	37.038	Constant
<0.001	12.04	0.427	0.015	0.186	ACI *
Step2					
<0.001	18.864		1.652	31.16	constant
<0.001	11.395	0.385	0.015	0.168	ACI *
<0.001	8.974	0.304	0.126	1.127	Usage hrs.

* Anxiety about COVID-19 Infection.

## Data Availability

Data of this research will be available on demand.
